# Medical Journalism as a Teacher

**Published:** 1883-06-20

**Authors:** 


### *MEDICAL JOURNALISM AS A TEACHER.*

The responsibility resting upon the teacher of any Science is so great, and the possible scope of his influence so boundless, the very greatness of the work makes it approach to the sublime. In this age of unprecedented progression and swift development of resources in so many of the arts that promise beneficence to the human race, no one can afford to be a laggard in the advanced movements of the period.

Especially should the teacher be enthused and energized in the race for the most honorable position, and which is attained when he worthily wins the guerdon of humanity's gratitude and reverence.

It has been said by some writer that to no body of men does the progressionist, who believes in the continual development of his race, look with more hope than to the members of the medical profession,'and that, taken as a whole, they lead the van in modern research—are foremost in accepting and frankly promulgating wider views in their profession, even though these views should operate for a season against their individual interest.

We of the profession must not disappoint this trustful attitude of the people towards us as teachers and ministers of medicine. We must indeed march to the tocsin of the times.

When we find "That a theory advanced by the apostles of medicine in the ages past, has fulfilled its purpose of nursing a germ of thought, we must drop the worn-out husks, and lay the foundation of higher achievements in medical science."

In doing this, the profession will not lose one jot nor tittle of its dignity and usefulness, but these will be enhanced by keeping pace with modern progression, and opening broader comprehensive fields for thought and action. We should endeavor to base our system upon a thorough knowledge, and the intelligent observance of nature's laws—upon a knowledge of man's organism, psycological as well as physical, and "the relation of his organism to his environments."

Progressive men of medicine are more closely observing the fact that disease is a disorder in the relation of the human organism to its surroundings, and that to restore harmony between these relations is the office of Medical Science.

A distinguished practitioner in England, in one of his lectures, speaks of the enlarged horizon of medical science in modern thought. He is fully armed with all information upon the subject, and observant of the fact that physicians are perceiving more clearly, day by day. a larger application of these principles of looking to the relations of man—as to what is around him in the fulfillment of the great purpose of preventing disease; and that it is in this direction the future of medicine lies clearly open—so to this end the progression must work.

The Congress of the United States has recognized this view in the organization of a National Board of Health. It is an appeal to medical science. It is an endorsement of its scientific status, of the wisdom and the power of the profession which we represent as members of its highest advisory bodies. Let us then, brethren, as journalists of American Medicine, resolve to do all in our power so as to satisfactorily respond to this appeal from the national halls of our great and glorious country.

We will keep this fact in view, that "Medicine will rise to the true heights of its great vocation when it watches over communities with able guardianship, and ministers to the race, as well as to the curing of disease in the individual man." In the possible attainments of Medical Science, "preventive medicine and measures must yet reach such a degree of perfection that the occurrence of epidemic diseases will be felt as a gross reproach to communities, and the physicians who preside over them."

This high attainment in Medical Science will naturally lead to the still higher and last step in its grand fulfillment of promise—that is, when it shall truly become a developer of the highest possibilities of the human race. It will then be a powerful moral agent, and "stand side by side with the minister of Christianity and the moral philosopher, and disclose and modify the deeper springs of human conduct, finding out the laws that have been at work in the origin and growth of good and evil," both of which have so great an influence upon the body, and the mental faculties of man, and whose study thus comes within the legitimate scope of Medical

Science. We shall then be able "to assist in carrying on the bettering process with more deliberate and intelligent methods and conscious purpose." But how shall medicine attain this fullness of measure?

Its teachers must labor for that end, each in his especial field of work. In no other way can it be acquired. We must be devoted to its pursuit, and patiently, with intelligent perseverance, search out and investigate every theory and truth of Medical Science as evolved by modern thought, then subject them to thorough analysis, and adapt them to intelligent practice.

The results of our action should then be given to the medical world through the pages of our professional literature.

The time is already almost at hand when a thinking and informed public will have no more uneducated manufactured quacks. They will demand that the physician shall be truly educated in head and heart, and we, as instructors in schools of medicine, or as mentors in medical literature, must teach the student and the practitioner of medicine, that "no course of instruction, no preparation for the stupendous work of his life can be too thorough."

Happily in this age of revolution in arts, literature, etc., Med'cal Science has caught the spirit of the times. It has made more intelligent progress in its many branches for the last twenty-five years, than in all the centuries before. This advancement is largely due to our professional journalism.

The circulation of medical literature throughout the civilized world has incited members of the profession to more deliberate study and higher aspirations in their field of labor.

It is very obvious that the theories and facts thus promulgated have awakened a train of more profound thought, and a more thorough investigation among medical men, and given them a thirst for still more knowledge of the great truths and principles of medicine. This we consider a most hopeful sign of what we believe will be future achievements in the profession, *en masse*, as well as in its distinguished leaders. T. S. P.

[Concluded in our next number.]

